# Developmental and/or Epileptic Encephalopathy with Spike-Wave Activation in Sleep: From Thalamocortical Mechanisms to Precision Therapy

**DOI:** 10.3390/children13080993

**Published:** 2026-07-27

**Authors:** Debopam Samanta

**Affiliations:** Division of Child Neurology, Department of Pediatrics, University of Arkansas for Medical Sciences, 1 Children’s Way, Little Rock, AR 72202, USA; dsamanta@uams.edu

**Keywords:** DEE-SWAS, ESES, CSWS, epileptic encephalopathy, spike-wave activation in sleep, thalamocortical network, precision medicine, Landau–Kleffner, corticosteroids, epilepsy surgery, L-serine, neurodevelopmental outcomes

## Abstract

**Highlights:**

**What are the main findings?**

• D/EE-SWAS is genetically heterogeneous, with monogenic causes found in up to one-third of patients, and these discoveries are increasingly enabling targeted, mechanism-based treatments such as L-serine for *GRIN* loss-of-function variants and primidone for *TRPM3*-related disease.
• Corticosteroids currently have the best-supported efficacy, though still with low-to-moderate certainty, for cognitive improvement; benzodiazepines are effective first-/second-line alternatives; and epilepsy surgery achieves SWAS resolution in roughly 80% of carefully selected patients—yet no drug has been formally approved and the evidence base rests mainly on small, retrospective studies.

**What are the implications of the main findings?**

• Clinicians should consider genetic testing early in the diagnostic workup of D/EE-SWAS, since it may uncover an actionable, etiology-specific treatment option and shift management from a syndrome-driven to a precision-medicine approach.
• Because current treatment evidence is of low to moderate certainty and highly heterogeneous, therapy should be individualized pragmatically while the field awaits adequately powered, controlled trials with standardized EEG and neurocognitive outcome measures.

**Abstract:**

Developmental and/or epileptic encephalopathy with spike-wave activation in sleep (D/EE-SWAS), previously described as continuous spike-wave during slow-wave sleep (CSWS) or electrical status epilepticus in sleep (ESES), is a childhood-onset disorder characterized by cognitive, language, behavioral, and/or motor regression or stagnation associated with marked activation of epileptiform discharges during non-rapid eye movement sleep. Three themes are central to the evolving understanding of D/EE-SWAS. First, it is a network disorder in which thalamocortical dysfunction, impaired sleep-dependent synaptic homeostasis, and potentially neuroinflammatory mechanisms contribute to neurodevelopmental deterioration. Second, increasing recognition of its genetic and structural heterogeneity is reshaping diagnostic evaluation. Monogenic etiologies are identified in up to one-third of cases, with a higher yield in the developmental and epileptic encephalopathy subtype, supporting early genomic testing alongside prolonged sleep EEG, MRI, and serial neuropsychological assessment. Third, treatment remains empiric and constrained by limited comparative evidence. Corticosteroids retain the strongest evidence base for cognitive improvement, although the overall certainty of this evidence remains low to moderate. Benzodiazepines are commonly used alternatives, and epilepsy surgery can provide substantial benefit in appropriately selected patients with focal structural abnormalities. Emerging etiology-directed therapies, including L-serine for selected *GRIN* loss-of-function variants and primidone for *TRPM3*-related disease, illustrate a broader transition from syndrome-based to precision management. However, no pharmacological therapy is specifically approved for D/EE-SWAS, long-term neurodevelopmental morbidity remains common, and adequately powered trials using standardized EEG and neurocognitive outcomes are urgently needed.

## 1. Introduction

Developmental and/or epileptic encephalopathy with spike-wave activation in sleep (D/EE-SWAS), formerly referred to as epileptic encephalopathy with continuous spike-wave during slow sleep (CSWS) or electrical status epilepticus in sleep (ESES), is a childhood-onset epileptic encephalopathy. It is characterized by cognitive, language, behavioral, and/or motor regression or stagnation, accompanied by marked activation of spike-and-wave discharges during non-rapid eye movement (NREM) sleep [1,2]. D/EE-SWAS accounts for approximately 0.2–1.3% of all pediatric epilepsies [2,3,4,5,6,7]. The earliest reported description of a similar condition with cognitive regression has been attributed to Kennedy and Hill’s 1942 report of “dementia infantilis with cortical dysrhythmia” [8]. In 1957, Landau and Kleffner described five children with acquired epileptic aphasia and diffuse interictal epileptiform discharges, with prominent temporal localization [9]. Sleep activation of epileptiform discharges was later described in 1971 by Patry, Lyagoubi, and Tassinari as subclinical electrical status epilepticus induced by sleep in six children [10]. In 1989, the ILAE reclassified it as CSWS, and in 2022 the ILAE Task Force on Nosology and Definitions proposed the current terminology to minimize confusion [1]: D/EE-SWAS for cases with pre-existing neurodevelopmental disorder, and EE-SWAS for cases with previously normal development who subsequently regress [1,11]. The umbrella term D/EE-SWAS encompasses both subtypes. Previously described atypical evolution of self-limited epilepsy with centrotemporal spikes, formerly termed atypical BECTS, and epileptic/acquired opercular forms characterized by drooling, dysarthria, speech arrest, orofacial-lingual weakness or dyspraxia, and impaired swallowing, may also fall within this syndrome when accompanied by SWAS on electroencephalography (EEG).

Beyond recent nomenclature refinements that have brought greater clarity to the clinical and EEG features of the syndrome, a growing number of genetic causes are now associated with D/EE-SWAS, some with potential for precision therapy. Concurrently, the convergence of structural and genetic etiologies is deepening our understanding of the pathophysiological mechanisms underlying D/EE-SWAS, positioning it as a valuable model for studying epilepsy-associated cognitive dysfunction—one of the most common and disabling comorbidities in epilepsy. Converging evidence implicates perisylvian network activation, thalamocortical dysfunction, and remote cortical inhibition, with complex interactions among cortical maturation, neuroplasticity, excitation–inhibition imbalance, and potential neuroinflammation driving the pathogenesis of D/EE-SWAS [12,13,14,15]. At the network level, an imbalance between excitatory glutamatergic dorsal thalamic neurons and inhibitory GABAergic reticular thalamic neurons has been proposed as the fundamental mechanism of SWAS [16,17]. Supporting a mechanistic link between nocturnal epileptiform activity and impaired offline memory consolidation, one study using simultaneous thalamic and cortical recordings demonstrated that sleep-activated cortical spikes propagate to the thalamus, prolong the thalamic spindle refractory period, and suppress spindle production for up to 30 s following thalamic spike discharge [18]. At the cellular level, this disruption manifests as an impairment of synaptic homeostasis (the synaptic homeostasis hypothesis, SHY). Under normal conditions, overnight NREM sleep produces a progressive decrease in slow-wave slope, reflecting net synaptic weakening that is essential for cognitive consolidation and synaptic renormalization. In D/EE-SWAS, spike-wave discharges abolish this overnight slope reduction at the epileptic focus, disrupting synaptic homeostasis and promoting aberrant synapse formation during critical developmental windows [19,20,21].

These exciting developments—spanning refined electroclinical characterization, expanding genetic landscapes, network-level insights, and emerging targeted therapies—underscore the need for a comprehensive and current review. This article aims to address that need, using a narrative synthesis of the literature conducted according to the methods outlined in Table 1.

## 2. Clinical and EEG Features

### 2.1. Clinical Presentation

The clinical spectrum of DEE-SWAS and EE-SWAS is characterized by seizure onset between 2 and 12 years of age, with a peak at 4–5 years. Both sexes are affected equally. Birth history is often normal, although premorbid neurological deficits and developmental delay may be present depending on the underlying etiology. In the initial phase, typically between 2 and 5 years of age, most patients have infrequent, drug-responsive focal motor seizures, with or without impaired consciousness, and focal to bilateral tonic–clonic seizures. Seizure onset is usually earlier in DEE-SWAS than in EE-SWAS. After 1–2 years from seizure onset, the EEG typically evolves to show characteristic spike-and-wave activation during sleep, associated with cognitive and/or behavioral regression or plateauing [22,23,24,25].

Regression in cognitive, behavioral, or psychiatric functioning is the cardinal feature of this syndrome, and clinical seizures may not be present or necessary for diagnosis [1]. Regression can fluctuate. All cognitive domains may be affected, including language and communication, temporospatial orientation, attention, executive function, learning/memory, and academic performance. Motor regression, including unilateral weakness (e.g., change in handedness), dyspraxia, ataxia, hyperkinesia, or dystonic features, may also occur [26]. Unusual manifestations include acquired visual agnosia [13]. Behavioral disturbances may include attention deficit, hyperactivity, aggressiveness, impaired social interaction, change in eating and sleeping habits, and, occasionally, autistic features [27]. In LKS (a subtype of D/EE-SWAS), the primary deficit is aphasia with an acquired auditory agnosia [1]. Pre-existing language or behavioral disorders are common in LKS prior to the onset of aphasia.

During this phase with regression, additional seizure types beyond the initial focal seizures may emerge, including typical and atypical absence seizures, atonic seizures, and focal motor seizures with negative myoclonus. Rare cases of absence status epilepticus have been reported at this stage, occasionally requiring intravenous therapy with agents such as midazolam or propofol. The presence of tonic seizures or epileptic spasms should prompt consideration of an alternative diagnosis.

Clinical seizures typically remit around puberty, even in patients with structural lesions. Neurocognitive and behavioral improvement is often observed; however, many patients have residual impairments that may be severe enough to limit independent functioning [23,28].

### 2.2. EEG Features

**Figure 1:**
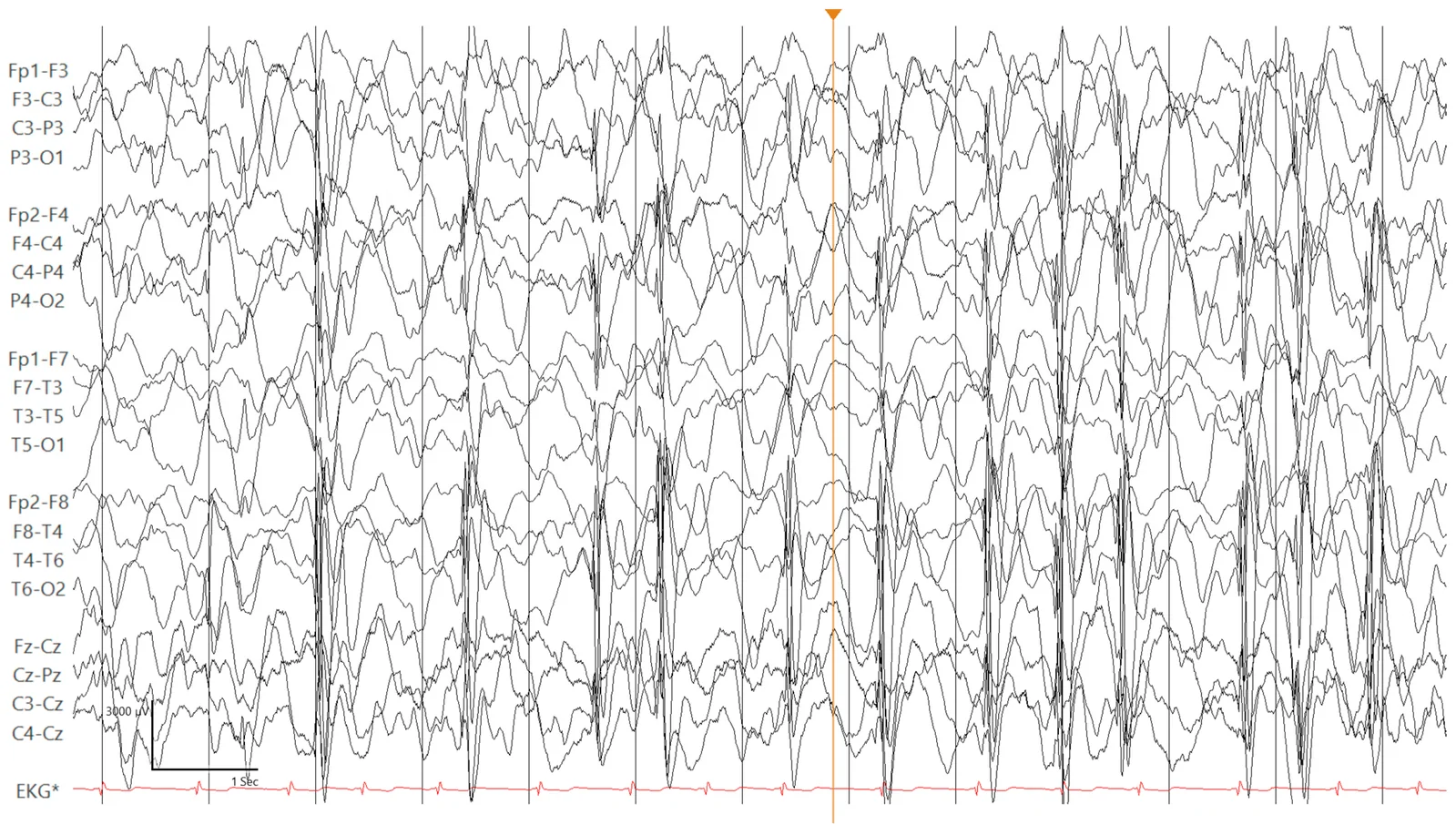
EEG showing diffuse activation of epileptiform discharges during sleep. 21-channel bipolar EEG recording, standard longitudinal anteroposterior (“double banana”) montage with parasagittal (Fp1-F3-C3-P3-O1, Fp2-F4-C4-P4-O2), temporal (Fp1-F7-T3-T5-O1, Fp2-F8-T4-T6-O2), and midline (Fz-Cz, Cz-Pz, C3-Cz, C4-Cz) chains, plus a single EKG channel. The tracing demonstrates a marked increase in diffuse, high-amplitude, bilaterally synchronous spike-and-slow-wave discharges during NREM sleep.

The hallmark EEG feature is marked activation of epileptiform activity during drowsiness and sleep, characterized by slow 1.5–2 Hz spike-and-wave complexes during NREM sleep; rarely, higher frequencies up to 3.5 Hz have also been reported [29]. SWAS is usually diffuse, but it may also be focal, often with frontal predominance, multifocal, regional, or lateralized over one hemisphere (Figure 1).

The diffuse spike-wave pattern may reflect synchronous bilateral spike activation, often with a 20–60 ms lead-in from one hemisphere, and a tangential or oblique dipole localizing to the perirolandic cortex [29]. Spikes may show both intraspike and interspike dipole instability, with changing dipole orientation likely reflecting local propagation around the cortical source and into the depth of the sulcus [29]. The clinical significance of the region of maximal continuous epileptiform activity amplitude—anterior versus posterior—or the broader distribution of epileptiform activity remains uncertain [30]. During REM sleep, these abnormalities become less frequent and may be absent. Normal sleep architecture, including vertex sharp waves, sleep spindles, and K complexes, is absent or difficult to distinguish. However, disturbed sleep architecture on EEG may have adverse cognitive consequences [31].

By contrast to the EEG during sleep, the waking background is variable: it may be normal, show focal or diffuse slowing, or contain focal or multifocal epileptiform abnormalities, but epileptiform discharges are typically much less prominent than during sleep.

The spike-wave index (SWI)—percentage of one-second bins occupied by at least one spike-wave during NREM sleep—is the primary quantitative metric for D/EE-SWAS diagnosis [32]. The original 85% threshold has been progressively supplanted; contemporary studies commonly use 50% as the diagnostic threshold, and current ILAE guidelines no longer mandate a specific percentage for diagnosis (neurobehavioral regression and improvement with steroids may occur in association with lower SWI) [1,33]. There is no consensus regarding the sleep stage (NREM vs. stages 3 and 4) or duration of sleep that should be used for scoring. Because quantifying SWI over an entire night is time-consuming, methods using the first 100 or 300 s of NREM sleep after the first sleep spindle have been reported [34,35,36]. Prior studies have varied in the SWI activation gradient from wakefulness to sleep, EEG duration, the number of sleep epochs analyzed, the choice of epochs analyzed (e.g., first epochs, first and last epochs, or selected NREM epochs), and epoch length [31,37,38,39]. An alternative metric, spike-wave frequency (SWF, spikes per unit time), avoids the ceiling effect intrinsic to SWI and may provide complementary information, though the relative superiority of SWF versus SWI for clinical correlation remains unresolved [24]. Some authors have suggested grading scales to quantify epileptiform discharges or evaluate various epileptiform and non-epileptiform activities [40,41]. Semi-automated or neural network-based automated SWI quantification systems are now available and perform much faster than expert visual scoring [24,42,43,44]. One study showed that higher gamma and ripple energy correlated with higher SWI, suggesting that high-frequency oscillations may track the severity of ESES [45].

D/EE-SWAS may rarely arise as the electroclinical evolution of self-limited epilepsy with centrotemporal spikes (SeLECTS) or self-limited epilepsy with autonomic seizures (SeLAS), though it may also follow other early-onset DEEs [16,46,47]. A SWAS pattern is frequently seen in these self-limited epilepsies, in which minor cognitive changes can occur and make differentiation from D/EE-SWAS difficult. Machine learning algorithms have been applied to resting-state EEG to predict which children will develop D/EE-SWAS [48]. Younger age at first seizure, higher pretreatment seizure frequency, and post-treatment seizure recurrence may be potential risk factors [49]. Two or more consecutive Rolandic spikes or polyspikes, particularly when accompanied by slow waves, may be associated with a higher sleep SWI [50]. In children with new-onset SeLECTS, higher sleep spike-wave burden (SWI ≥ 50%) was associated with worse WISC-IV cognitive performance and band-specific under-representation of MEG source activity in cognitive network regions, particularly alpha-band medial frontal cortex and beta-band posterior cingulate cortex activity [51]. The language effects may be subtle, with impaired complex sentence formulation and word production that may not be captured by standard objective testing but can be detected using narrative assessment tools, particularly through deficits in complex syntactic production [52]. In summary, a SWAS pattern without cognitive or behavioral regression should not be diagnosed as D/EE-SWAS. However, careful serial clinical and neuropsychological assessment is needed to avoid missing evolving D/EE-SWAS.

## 3. Diagnostic Evaluation

### 3.1. EEG

Overnight video-EEG polysomnography is the diagnostic gold standard. In practice, prolonged ambulatory EEG and nap recordings are also used [53,54,55]. A wearable two-channel EEG device with semi-automatic spike quantification has demonstrated utility for serial monitoring of interictal activity [42]. Serial EEGs are recommended every 1–6 months to assess treatment response. Emerging monitoring tools include subcutaneous EEG for continuous ambulatory surveillance—still investigational but potentially valuable for dynamic treatment monitoring [12]. One study reported an individualized hybrid expert system using biogeography-based optimization to automate ESES quantification from EEG, achieving low estimation error in 20 children and suggesting that patient-specific machine-learning approaches may support more reproducible SWI assessment [56]. Using difference visibility graph-based analysis of NREM sleep EEG, one study reported higher degree entropy in patients with D/EE-SWAS, particularly over central and left parietal regions, supporting its potential utility as a quantitative biomarker [57]. Efforts to identify biomarkers in high-risk children suggest that spike frequency and delta power may help predict D/EE-SWAS development after perinatal stroke [58].

### 3.2. Neuroimaging

High-field (3T preferred) brain magnetic resonance imaging (MRI) is essential in all patients to identify structural lesions associated with D/EE-SWAS, including thalamic lesions from perinatal hypoxic–ischemic injury, thalamic hemorrhage, polymicrogyria, focal cortical dysplasia, schizencephaly, porencephaly, and hydrocephalus [59,60,61,62,63]. Reduced relative thalamic volume has been demonstrated even without MRI-visible lesions [64,65,66]. One study reported advanced MRI analyses and found altered thalamic diffusivity, increased temporal cortical thickness, and additional white matter and subcortical volume changes in genetically positive patients, suggesting involvement of corticothalamic, corticostriatal, and cerebellar networks [67]. Functional neuroimaging has further illuminated the network-level consequences. 18F-fluorodeoxyglucose positron emission tomography (FDG-PET) consistently demonstrates hypermetabolism in perisylvian, superior temporal, inferior parietal, and central cortical areas corresponding to epileptic foci, alongside hypometabolism in default mode network (DMN) regions (precuneus, posterior cingulate cortex, prefrontal cortex, parahippocampus) and thalamus attributable to remote inhibition [68,69,70]. A greater number of hypometabolic regions may be associated with a worse behavioral profile [68]. EEG-fMRI studies demonstrate positive BOLD signal changes in perisylvian regions, prefrontal cortex, anterior cingulate, and thalamus, with negative BOLD in DMN regions—a pattern independent of etiology [15,71]. In an fMRI study, lower WISC-IV FSIQ and perceptual reasoning scores were accompanied by altered resting-state ReHo across frontal, sensorimotor, cerebellar, precuneus, and Rolandic-opercular regions, suggesting disrupted local network synchrony as a potential neurofunctional correlate of cognitive impairment [72].

When conventional MRI is normal but focal onset is suspected, advanced techniques including FDG-PET, ictal single-photon emission computed tomography (SPECT), and magnetoencephalography (MEG) are indicated, particularly for pre-surgical evaluation—these may reveal hidden focal cortical dysplasia as the underlying cause [73,74].

### 3.3. Genetic Testing

Early studies from the 1990s identified predominantly structural causes in 30–60% of cases, while etiology remained unknown in a substantial proportion of patients, approximately 40–70% [75,76]. Genetic causes were essentially unrepresented in these earlier cohorts. By 2009, structural causes were recognized in approximately 40% of cases while genetic causes were still largely absent from series [77]. The landscape shifted dramatically with the advent of next-generation sequencing. The first gene specifically related to SWAS was *GRIN2A*, identified in 2013 in 9% of patients with the epilepsy-aphasia spectrum [78]. Subsequent studies revealed that many variants previously associated with other DEEs—including *SCN2A*, *KCNA2*, *KCNQ2*, *KCNQ3*, *SLC9A6*, *CNKSR2*, *ZEB2*, *SHANK3*, *KMT5B*, *CHD2*, *CDKL5*, and *SYNGAP1*—are also associated with D/EE-SWAS [12,79]. In a cohort of 52 patients, one study identified *GRIN2A* variants in 15.5% of cases and additional likely pathogenic variants in GABBR2, *SCN1A*, *TRPC1*, *ERRFI1*, *CTXN3*, *IRX6*, and *IQCA1* [80]. The identification of DLG4-related synaptopathy as causing D/EE-SWAS in more than 25% of affected individuals is a recent significant finding [81].

Contemporary studies report genetic etiologies in 10–37% of cases [82,83,84,85,86,87]. A recent study reported that genetic causes are far more prevalent in DEE-SWAS (55%) than in EE-SWAS (15%), suggesting that pre-existing developmental impairment warrants comprehensive genetic evaluation as a priority [85]. Whole exome or genome sequencing (WES/WGS) is the preferred first-tier genetic test when MRI is non-informative; chromosomal microarray (CGH-array) should be performed when there is clinical suspicion of 17q21.31 and 15q13.3 deletions or Xp11.22–p11.23 duplication [79]. Some structural causes of D/EE-SWAS may have identifiable genetic etiologies, including mTOR-pathway variants in focal cortical dysplasia type II and polymicrogyria associated with 22q11.2 deletion, 1p36 deletion, or variants in *GPR56*, *TUBB2B*, *SRPX2*, *PAX6*, *EOMES*, *WDR62*, *TUBA8*, *KIAA1279*, and *COL18A1* [88,89]. Cases with normal or uncertain genetic results should undergo periodic reanalysis and consideration of whole genome sequencing, RNA sequencing, and epigenetic studies [12].

### 3.4. Neuropsychological Assessment

Standardized serial neuropsychological testing is indispensable but chronically underutilized in clinical practice. Among 28 studies reporting cognitive outcomes in a systematic review, only 12 used validated instruments—including the Wechsler Intelligence Scales, Strengths and Difficulties Questionnaire, and Terman–Merrill Scales [90]. The remainder relied on subjective parental or clinician reports or did not define the assessment method. Language assessment in non-native speakers presents an additional challenge [91]. Serial evaluations using standardized instruments at baseline and at 6-month intervals are strongly recommended, covering domains of language, attention, executive function, motor skills, and adaptive behavior. The frequency of cognitive testing is a barrier in clinical practice due to time and insurance coverage limitations [92]. Testing at very short intervals may also introduce test–retest bias, as improved performance may reflect familiarity with the test rather than true cognitive change.

### 3.5. Additional Investigations

**Figure 2:**
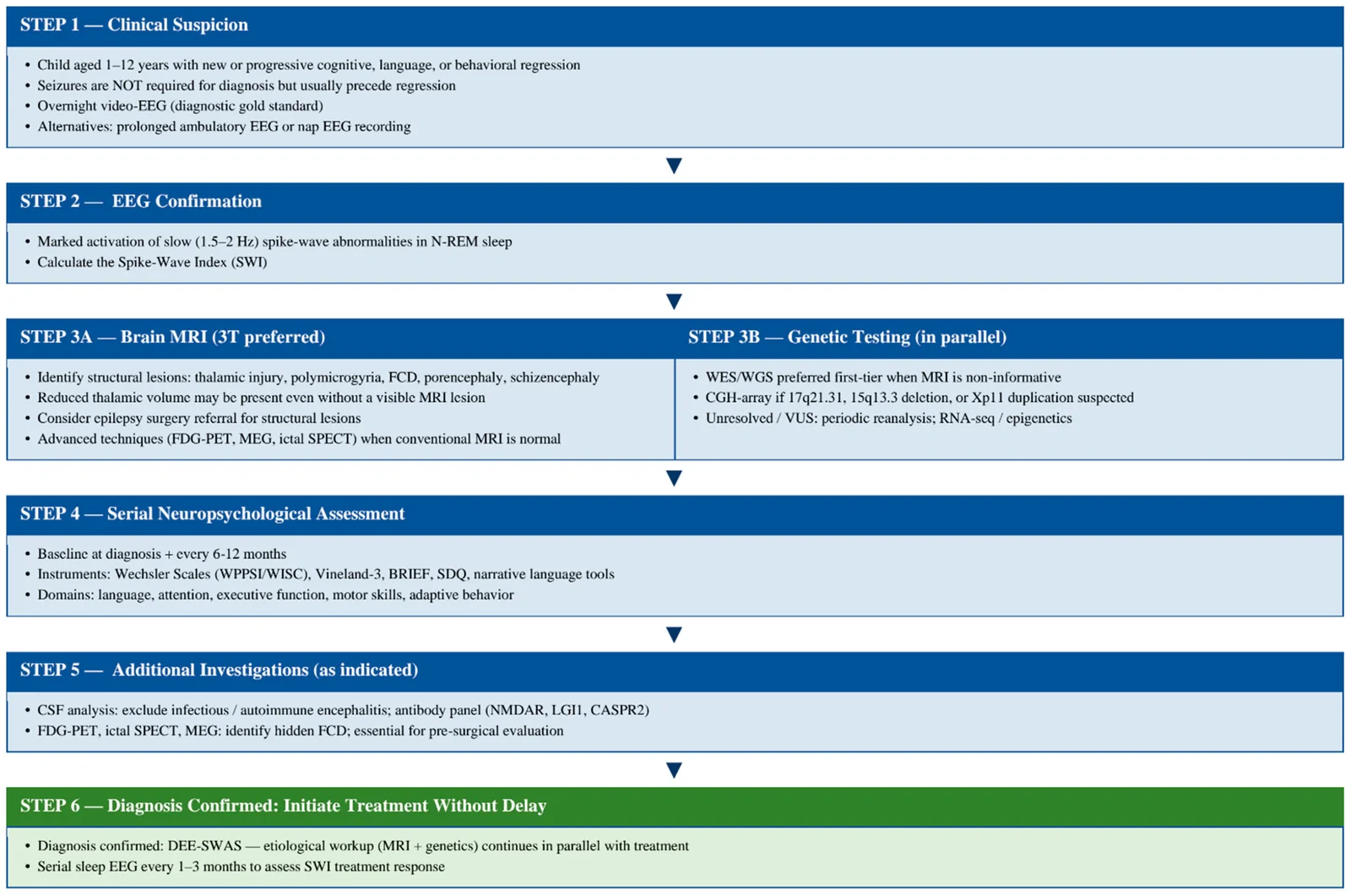
Recommended Diagnostic Algorithm for DEE-SWAS. Abbreviations: CGH = comparative genomic hybridization; CSF = cerebrospinal fluid; EEG = electroencephalogram; FCD = focal cortical dysplasia; FDG-PET = fluorodeoxyglucose PET; MEG = magnetoencephalography; N-REM = non-rapid eye movement; SWI = spike-wave index; VUS = variant of uncertain significance; WES/WGS = whole exome/genome sequencing.

Cerebrospinal fluid analysis is indicated to exclude infectious and autoimmune encephalitis; onconeuronal and neuronal surface antibody panels should be obtained where indicated [93]. One study reported elevated cerebrospinal fluid levels of IL-1β, TNF-α, IL-1α, and caspase-1 in D/EE-SWAS [94]. Serum inflammatory markers—particularly interleukin-6 and 8, which correlates with D/EE-SWAS disease activity and decreases with immunomodulatory treatment—may serve as a supplementary biomarker [95,96]. One study reported reduced native and total thiol levels, increased ischemia-modified albumin, and higher disulfide/native thiol ratios in children with D/EE-SWAS compared with controls, suggesting a shift toward systemic oxidative stress [97]. Thiol levels negatively correlated with SWI, although these findings require validation before clinical use as biomarkers. Another study reported no change in serum neuron-specific enolase in D/EE-SWAS compared to control subjects, suggesting no neuronal cell loss from interictal activity [98]. Figure 2 presents the recommended diagnostic algorithm.

## 4. Differential Diagnosis

D/EE-SWAS must be distinguished from several other conditions. Lennox–Gastaut syndrome shares cognitive regression and drug-resistant epilepsy but is distinguished by tonic seizures, slow spike-and-wave on waking EEG (<2.5 Hz), and paroxysmal fast activity during sleep [99]. Uncomplicated SeLECTS is readily differentiated by the absence of cognitive regression and SWI typically below 50% on sleep EEG. Drug-induced D/EE-SWAS—precipitated by carbamazepine, oxcarbazepine, phenytoin, and phenobarbital—resolves with medication withdrawal and must be excluded before embarking on immunotherapy [100]. Autoimmune encephalitis, particularly NMDAR encephalitis, may produce EEG patterns mimicking D/EE-SWAS and can be excluded with a comprehensive antibody panel [93]. DEE–not otherwise specified may be considered when a SWAS pattern is present without clear developmental regression, particularly in individuals with baseline intellectual disability or autism and a suspected genetic etiology.

## 5. Treatment

No pharmacological agent is currently approved for D/EE-SWAS, and the treatment evidence base consists almost entirely of retrospective case series and a handful of small prospective trials [27,101,102,103,104,105,106,107,108,109,110,111,112,113,114,115,116,117,118,119,120,121,122,123,124,125,126,127,128,129,130,131,132,133,134,135,136,137,138,139,140,141,142,143] (Table 2).

**Figure 3:**
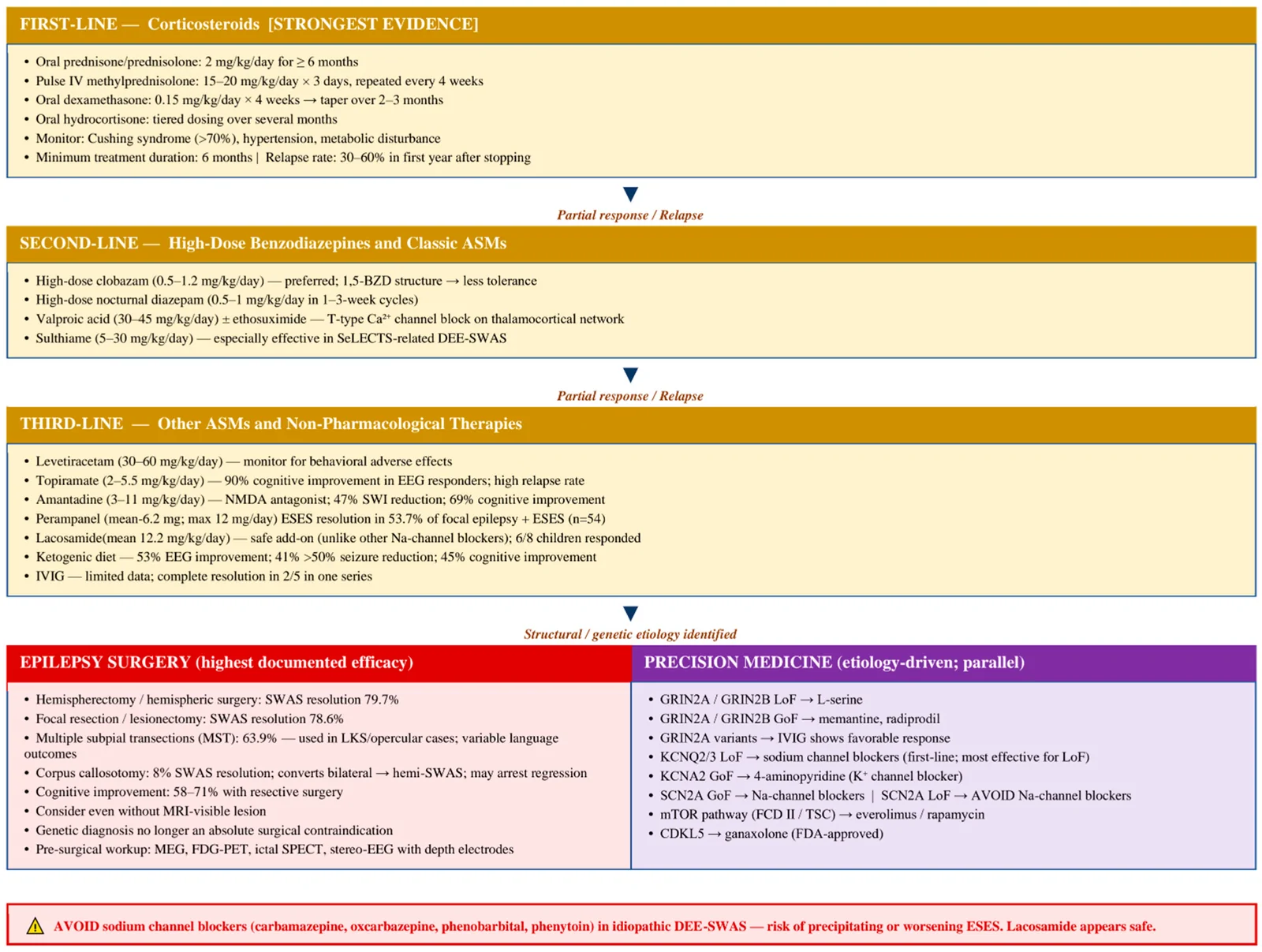
Proposed treatment algorithm for DEE-SWAS: syndrome-driven + etiology-driven approach. Abbreviations: ASM = antiseizure medication; CDKL5 = cyclin-dependent kinase-like 5; ESES = electrical status epilepticus during sleep; FCD = focal cortical dysplasia; GoF = gain of function; IVIG = intravenous immunoglobulin; LKS = Landau–Kleffner syndrome; LoF = loss of function; MEG = magnetoencephalography; NMDA = N-methyl-D-aspartate; SWAS = spike-wave activation in sleep; SWI = spike-wave index; TSC = tuberous sclerosis complex.

Taken together, the studies summarized in Table 2 suggest a rough hierarchy of treatment response across modalities, tempered throughout by small sample sizes, retrospective designs, and heterogeneous outcome definitions. Corticosteroids show the most consistent cognitive benefit and rest on the largest aggregate patient numbers, though relapse after taper is common. Benzodiazepines, particularly diazepam and clobazam, produce meaningful spike-wave index reduction but are generally positioned as first- or second-line adjuncts rather than substitutes for steroids, as illustrated by the RESCUE ESES trial. Other antiseizure medications (levetiracetam, valproate, sulthiame, and others) and the ketogenic diet offer modest, variably reported benefit and are most useful within combination regimens. Neuromodulation data remain the most limited, drawn almost exclusively from case reports and small series, and should be interpreted cautiously. Epilepsy surgery achieves the highest reported rates of SWAS resolution, but these outcomes come from highly selected, non-randomized surgical cohorts and are not directly comparable to outcomes with medical therapy. Overall, no single therapy is curative, relapse is frequent across modalities, and the certainty of the underlying evidence remains low to moderate. Treatment duration and sequence outcome measures used for response assessment, including EEG, seizure, and cognitive endpoints, relapse rates, and short- versus long-term outcomes, are heterogeneously reported, making cross-study comparisons challenging (Figure 3).

### 5.1. Corticosteroids, ACTH, and IVIG

Given the proposed inflammatory or immune-mediated mechanism, corticosteroids, adrenocorticotropic hormone (ACTH), and intravenous immunoglobulin (IVIG) have been used [40,144]. Corticosteroids represent the treatment of choice based on the strongest available evidence, recommended as first-line therapy either alone or in combination with ASMs, including benzodiazepines [86,100,136,145,146,147]. A pooled analysis of 575 patients demonstrated steroid-associated clinical or EEG improvement in 75–81% of cases, significantly outperforming ASMs alone (49%) and benzodiazepines (68%), with surgery achieving the highest rates at 90% [141]. The landmark RESCUE ESES randomized controlled trial compared steroids versus clobazam as primary outcomes for cognitive function at 6 months; data favored early steroid use (an improvement of >11.25 IQ points was noted in 5 out of 25 (25%) children) [118]. However, this change was not reflected in the cognitive sum score. Changes in SWI, EEG responder rate, defined as a ≥25% reduction in SWI from baseline, and the proportion of patients achieving SWI < 50% did not differ significantly between groups at either 6 or 18 months. In a 10-center retrospective cohort of 72 children with D/EE-SWAS, initial corticosteroid therapy (n = 24) was associated with greater improvement in daily functioning at 6 months than clobazam (84% vs. 51%) and greater SWI reduction, with both treatments generally well tolerated and intravenous methylprednisolone better tolerated than daily oral prednisolone [127]. In a prospective cohort of 39 patients treated with corticosteroids or IVIg, SWI improved from 80% (65–91) to 37% (24–65) over 12 months, alongside improvements in psychiatric symptoms, language function, communication, and disability [148]. Later onset and concomitant benzodiazepine therapy may be associated with higher steroid response rates, whereas earlier onset and frontal epileptiform discharges may increase the risk of recurrence after steroid therapy [149].

Multiple steroid formulations and regimens have been used: oral prednisone/prednisolone (2 mg/kg/day), pulse intravenous methylprednisolone (15–20 mg/kg for 3 days followed by oral prednisolone or repeat pulse dose every 4 weeks), oral dexamethasone (0.15 mg/kg/day orally for 4 weeks, followed by 2–3 months of maintenance and gradual taper in responder), and hydrocortisone (5 mg/kg/day for 1 month, then 4 mg/kg/day in month 2 and 3 mg/kg/day in month 3; if EEG/clinical response, tapered to 2 mg/kg/day for 9 months, then slowly tapered off over a total treatment duration of 21 months) [114,118,126]. Prospective open-label studies of combined methylprednisolone plus prednisolone and oral dexamethasone each reported approximately 47–50% of patients achieving meaningful seizure reduction, SWI improvement, and cognitive gains [115,126]. A minimum of 6 months of treatment is generally recommended, with no consensus on the optimal regimen. Relapse rates are high: 29–59% within the first year [116,150]. Major adverse effects—Cushing syndrome (reported in >70% in some series), hypertension, and metabolic disturbance—require careful monitoring [115,126]. To mitigate cushingoid adverse effects, one study reported successful use of weekly oral prednisone at 2 mg/kg/week for a mean duration of 10 months [117]. Deflazacort, an oxazoline derivative of prednisolone with a potentially more favorable metabolic profile, was used in two patients in one study, both of whom were seizure-free at 6 months [151].

Reports of ACTH use are rare. ACTH produced 50–59% SWI reductions and seizure freedom in 57–70% of patients with seizures in two retrospective studies, with notable improvements in ADHD/ASD-like symptoms [27,125,152]. ACTH was administered at 0.03 to 0.06 mg/kg/day, with treatment duration based on SWI severity: 6 to 10 days for SWI of 15% to 30%, and 12 to 15 days for SWI above 30%. Patients received an average of 3 to 4 repeated treatment courses. Speech recovery occurred in only a minority (12–16%) of patients.

Reports on IVIG use are scarce [153,154,155]. In a small open-label series of five patients, IVIG was associated with significant short-term clinical and EEG improvement, including complete symptom resolution in two patients, but efficacy remains uncertain given the uncontrolled design and small sample size [40].

Other immunomodulatory agents, including anakinra, an interleukin-1β inhibitor, and sirolimus, have been associated with improvements in cognition, speech, and behavior in anecdotal reports [156,157].

### 5.2. Benzodiazepines

High-dose benzodiazepines—particularly clobazam (0.5–1.2 mg/kg/day) and diazepam (0.5–1 mg/kg/day in short cycles of 1–3 weeks)—are commonly used as first- or second-line therapy and are more effective than other ASMs [45,132,158,159,160]. A first-night oral diazepam dose of 1 mg/kg is still used to assess EEG spike-wave suppression, whereas rectal administration is no longer routinely employed [117,161]. A series of 29 patients receiving high-dose diazepam demonstrated a mean SWI decrease of 36% [107]. Retrospective data from three diazepam studies reported SWI reductions of 47–65% from baseline, with improvements in language (48%), cognition (38%), and behavior (14%) [107,108]. In a prospective cohort of 18 children, high-dose nocturnal diazepam (0.5 mg/kg/d, maximum 15 mg) for 6 months reduced mean sleep SWI from 85.7% to 32.6% and aggregate sleep EEG score from 6.5 to 3.1, although EEG relapse occurred in 10 children and mild side effects in 5 [162]. Clobazam (response rate 33–73%) use has increased relative to diazepam in recent years, likely because its 1,5-benzodiazepine structure may allow easier chronic use with less concern for tolerance [163]. The RESCUE ESES trial directly compared steroids with clobazam with data favoring steroids; benzodiazepines are thus best positioned as second-line agents or adjuncts. Relapse is common after benzodiazepine discontinuation, and adverse effects including irritability and behavioral disinhibition require monitoring [139]. Other benzodiazepines, including clonazepam, lorazepam, midazolam, and nitrazepam, have been used only rarely [132,133,164,165].

### 5.3. Levetiracetam

Levetiracetam is often considered a preferred ASM for D/EE-SWAS, although careful monitoring is needed because of potential behavioral adverse effects. Two RCTs of levetiracetam (20–25 mg/kg, 12-week crossover design) demonstrated significant SWI reductions versus placebo (40% vs. 17% and 44% vs. 8%) but neither assessed seizure frequency or demonstrated cognitive benefit [105,120]. Higher-dose retrospective studies (30–60 mg/kg) reported SWI normalization in 21–34%, seizure freedom or reduction in 63–70%, and cognitive recovery in a minority [106,124]. Brivaracetam, which also binds synaptic vesicle protein 2A (SV2A), may be a suitable alternative for some patients because of its more favorable behavioral tolerability profile [166].

### 5.4. Valproic Acid and Ethosuximide

Valproic acid (30–45 mg/kg/day), alone or in combination with ethosuximide or clobazam, has demonstrated 43–65% response rates in seizure control, cognitive improvement, and EEG suppression in small series [102,103,104]. It remains a useful drug in multi-agent regimens. Ethosuximide blocks T-type calcium channels in thalamic neurons and theoretically exerts effects on the thalamocortical epileptic network in D/EE-SWAS; it is most commonly used in combination with valproic acid or benzodiazepines [167]. One study suggested that a higher pretreatment modulation index—reflecting stronger slow-wave coupling with gamma or ripple oscillations across wider brain regions—may identify a thalamocortical network phenotype associated with better response to ethosuximide [167].

### 5.5. Sulthiame

Sulthiame (a sulfonamide carbonic anhydrase inhibitor, 5–30 mg/kg/day) is particularly effective as an add-on therapy in D/EE-SWAS arising from SeLECTS, with reported responder rates of 60–75% in this subgroup [122]. Retrospective studies reported EEG normalization in 24–28% of patients, seizure freedom or reduction in 58–63%, and cognitive improvement in 38% [111,122,123]. Its use is limited by significant adverse effects: hyperventilation/agitated respiration (6–14%), ataxia, headache, anorexia, and metabolic acidosis [90].

### 5.6. Other ASMs

Several other ASMs have been evaluated in a limited number of studies. Topiramate (2–5.5 mg/kg/day) improved EEG in 48% and produced cognitive/behavioral improvement in 90% of EEG responders in 21 patients, with high relapse rates [109]. Amantadine (NMDA antagonist, 3–11 mg/kg/day) reduced median SWI by 47% and produced language, cognitive, or behavioral improvement in 69% of 20 patients with refractory ESES over a median 11.5 months [112]. Another study showed EEG normalization and seizure control in 4 of 5 patients (80%) [168]. Perampanel (AMPA antagonist) resolved ESES in 53.7% of 54 children with focal epilepsy and ESES in one retrospective study [169]. Lacosamide add-on achieved responder status in 6/8 children after 6 months [110], and acetazolamide produced CSWS resolution in 3/6 patients with ESES or LKS [170]. Felbamate was associated with clinical and electrographic improvement in a cohort of 22 patients, including 16 without seizures and 6 with seizures: 4 of 16 seizure-free patients had complete EEG resolution with 100% reduction in spike-wave index, while 2 of 6 patients with seizures achieved seizure resolution [171]. In a small open-label exploratory study of six children with DEE-SWAS, fenfluramine was well tolerated over a median 12-month follow-up, with SWI improvement in 4/6 patients (66.7%), including ≥50% SWI reduction in 2/6 and near-resolution in one, while concomitant corticosteroid and antiseizure medication burden was reduced in 4/6 patients (66.7%) [172]. Executive functioning also showed a trend toward improvement in 4/6 patients (66.7%) based on BRIEF-2 scores, while Vineland-3 scores remained stable and caregivers reported perceived improvements in behavior, cognition, and sleep [172].

Sodium channel blockers (carbamazepine, oxcarbazepine, phenobarbital, phenytoin) should be avoided in idiopathic D/EE-SWAS due to risk of precipitating or worsening ESES, though lacosamide appears to be a safe exception [12].

### 5.7. Ketogenic Diet

A review of six studies (1999–2016) covering 38 children with D/EE-SWAS treated with the ketogenic diet (KD) found EEG improvement in 53%, seizure reduction > 50% in 41%, and cognitive improvement in 45%, with EEG normalization in only 9% [119]. The KD may exert its effects in D/EE-SWAS partly through influence on GABA neurotransmission and anti-inflammatory mechanisms [173]. Combination KD plus oral steroids enabled steroid discontinuation in 8/13 steroid-dependent patients in one monocenter study [174]. In some genetic etiologies, such as Koolen–de Vries syndrome, dietary therapy may be particularly beneficial [175].

### 5.8. Neuromodulation

Data on neuromodulation in D/EE-SWAS remain very limited. Because the available literature is drawn almost entirely from isolated case reports and very small case series, the observations below should be regarded as preliminary and hypothesis-generating rather than as evidence of established therapeutic benefit, and are presented as illustrative examples rather than practice recommendations pending confirmation in larger, controlled studies.

Vagus nerve stimulation (VNS) showed a 50% seizure response rate in 6 patients in one published study [176]. In another case report, a 12-year-old child became seizure-free, with complete resolution of SWAS and significant cognitive improvement following VNS therapy [177]. Deep brain stimulation (DBS)—routinely used in Lennox–Gastaut syndrome targeting the centromedian nucleus— has not been formally evaluated in D/EE-SWAS; its hypothetical thalamic targeting could be beneficial in super-refractory cases, but clinical data are absent [178,179,180,181,182]. Transcranial direct current stimulation (tDCS) remains investigational [183]. One study reported that simultaneous MEG-guided tDCS–rTMS was safe and associated with improved language function in two of three patients, with MEG connectivity suggesting enhanced bilateral language-network information flow [184]. An ongoing clinical trial (NCT04034030) is evaluating repetitive transcranial oscillatory magnetic stimulation (rTOMS) in D/EE-SWAS patients. In another study of nine patients, 10 days of low-frequency rTMS or continuous theta burst stimulation reduced SWI, increased sleep spindle density and spindle–slow wave coupling, left 75% seizure-free at 6 months, and was associated with improved IQ and mental age [185].

### 5.9. Epilepsy Surgery

Surgery achieves the highest documented treatment efficacy in D/EE-SWAS. A recent systematic review and meta-analysis reported SWAS resolution rates of 79.7% for hemispherectomies, 78.6% for focal resections, 63.9% for multiple subpial transections (MST; parallel, shallow cortical transections are made to interrupt horizontal intracortical epileptiform propagation while preserving vertical columnar pathways and thereby reducing risk to language or motor function), and only 8.3% for corpus callosotomy [142].

Resective surgeries in structural lesion patients (hemispherectomy/lesionectomy) produced seizure and SWAS resolution in 77–80% and cognitive improvement in 58–71% [186,187]. Importantly, D/EE-SWAS may be surgically remediable even without MRI-visible lesions [73], and a genetic diagnosis no longer represents an absolute surgical contraindication. MST may improve seizures, EEG abnormalities, and behavior, but language outcomes are variable, cognitive outcomes are poorly characterized, comparative benefit over nonsurgical management is unproven, and reported complications include cerebral edema and transient neurological deficits [188,189]. The use of MST has declined at many centers as more precise resective and neuromodulatory techniques have become available. Corpus callosotomy, while rarely achieving SWAS resolution (8%), commonly converts bilateral to hemispheric SWAS and may arrest cognitive regression [190]. Pre-surgical workup should include MEG, FDG-PET, ictal SPECT, and stereo-EEG with depth electrodes as necessary [12].

These favorable surgical outcomes derive almost entirely from retrospective cohorts of highly selected patients and are therefore not directly comparable to outcomes with medical therapy, given the substantial selection bias inherent in surgical case series. Surgical morbidity, including hemiparesis (particularly after hemispherectomy), visual field deficits, and other focal neurological deficits, must be weighed against these outcomes. MRI-negative epilepsy surgery in particular requires highly specialized, multimodal presurgical evaluation and is generally reserved for carefully selected patients with concordant electrophysiological or functional imaging findings.

### 5.10. Precision Medicine: Etiology-Driven Treatment

The most significant recent development in D/EE-SWAS management is the emergence of precision medicine approaches guided by specific genetic diagnoses [12]. These strategies represent a fundamental shift from the traditional syndrome-driven paradigm and encompass optimizing known ASM indications, repurposing existing medications, and developing new targeted agents.

GRIN2A-related D/EE-SWAS, the most common monogenic cause (up to 20% of epilepsy-aphasia spectrum cases), exemplifies this approach [191]. Pathogenic *GRIN2A* variants produce either gain-of-function (GoF) or loss-of-function (LoF) of the NMDA receptor GluN2A subunit [191]. For LoF variants, NMDA receptor co-agonist supplementation with L-serine is a promising strategy: a Phase 2A non-randomized open-label trial in 18 patients with GRIN LoF (*GRIN1*, *GRIN2A*, *GRIN2B*) demonstrated improvements in adaptive behavior, motor function, and quality of life, with complete resolution of epileptic activity in 5/18 (28%) and seizure frequency reduction in 1/3 patients with seizures (75–90% responder rate) [143]. For GoF variants, NMDA receptor antagonists—memantine and radiprodil (preclinical GRIN2A DEE mouse models and clinical trial)—are the targeted approach [191,192]. Patients with *GRIN2A* mutations also show favorable responses to IVIG [193]. Additionally, two children with *TRPM3* gain-of-function-associated D/EE-SWAS showed resolution of SWAS, cessation of developmental regression, and psychomotor improvement following treatment with primidone, which inhibits TRPM3 activity [194]. Additional potential precision-therapy options are summarized in Table 3.

However, these targeted approaches are applicable to only a small, genetically defined subset of patients, and reported efficacy is drawn largely from genotype-specific cohorts (e.g., *GRIN*-related or *TRPM3*-related disease) rather than from the D/EE-SWAS phenotype as a whole; genetic testing results should therefore be interpreted with this distinction in mind.

## 6. Outcomes

### 6.1. Seizure Outcomes

Seizure outcomes in D/EE-SWAS are generally more favorable than neurodevelopmental outcomes [140]. The natural history tends toward seizure remission by adolescence in most patients, mirroring the self-limiting trajectory of related childhood focal epilepsies. Seizure freedom rates at last follow-up range from 38% to 90% across observational studies, with higher rates in longer follow-up studies and in non-lesional etiology [128,129,130]. Treatment-related seizure control ranges from 47 to 100% across drug evaluation studies but varies substantially by agent and population [90,122].

### 6.2. Neurodevelopmental Outcomes

Neurodevelopmental outcomes are frequently unsatisfactory; complete cognitive recovery after EEG remission occurs in only a minority of patients, and up to half are left with deficits that limit independent functioning [23,28,38,195]. Disruptive behaviors and poor adaptive functioning may contribute to reduced health-related quality of life; structured psychological interventions, including parent training, may help address these challenges [196]. For LKS, complete language recovery occurs in approximately 28% at 12-year follow-up [130].

The most consistently identified adverse prognostic factors are longer ESES duration and younger age at onset—both associated with more severe and enduring cognitive and language deficits across multiple studies [39,124,128,130]. Additional unfavorable factors include structural lesions, slow EEG background, high SWI, drug resistance, and polypharmacy [39,197,198]. Favorable prognostic factors include older age at onset, shorter D/EE-SWAS duration, normal MRI, normal development before encephalopathy, SeLECTS phenotype at onset, late-onset aphasia, and early EEG normalization with treatment [28,141,199] (Table 4).

Sleep slow-wave slope analysis has emerged as a biomarker of cognitive trajectory: impaired overnight slope decrease during ESES at the epileptic focus correlates with worse cognitive outcomes, while post-remission normalization of slope predicts cognitive recovery [20,200]. HFOs may provide steroid-responsive prognostic information in D/EE-SWAS, appearing more treatment-sensitive than spikes, with greater steroid-related reduction in genetic or unknown etiologies than in structural etiologies, while persistent HFOs after steroid therapy have been associated with seizure relapse and unfavorable cognitive outcomes [115,135]. Several studies associate EEG improvement with subjective cognitive or IQ and language gains, while others—particularly smaller studies with shorter follow-up—find no such correlation [90,117,130,137,138].

## 7. Future Research

The field of D/EE-SWAS is at an inflection point: decades of predominantly syndrome-driven, low-quality evidence are giving way to a precision medicine era anchored in genetic diagnosis and targeted therapeutics. Several priority areas define the research agenda.

First, adequately powered randomized controlled trials with prespecified EEG, seizure, and standardized neurocognitive endpoints remain urgently needed, including sensitive outcome measures beyond IQ, which may not change meaningfully in the short term. Several recently completed or ongoing trials are promising, including RESCUE ESES comparing corticosteroids with clobazam (ISRCTN42686094), cannabidiol versus placebo (NCT04721691), fenfluramine in DEEs including D/EE-SWAS (NCT05232630), the phase 2 NBI-827104 T-type calcium channel blocker study and open-label extension (NCT04625101/NCT05301894), and rTOMS (NCT04034030). However, additional rigorously designed studies are still needed.

Second, the phase 2A L-serine trial in GRIN loss-of-function disorders, which included one patient with D/EE-SWAS, demonstrates the feasibility of genotype-guided therapy and provides a template for future precision-medicine trials [143]. Drug repurposing—applying existing agents with new mechanistic rationale—offers a faster pathway than de novo drug development.

Third, consensus on standardized diagnostic criteria (agreed SWI thresholds, calculation methods) and a validated international neuropsychological assessment battery are prerequisites for meaningful cross-study comparability [1].

Fourth, novel biomarkers—including HFOs, sleep slow-wave slope analysis, interleukin-6, and MRI-based thalamic volumetrics—may supplement SWI as dynamic treatment monitoring tools and prognostic indicators. Using predictive biomarkers to identify at-risk children before the onset of D/EE-SWAS and to guide preventive therapy may represent the next frontier [58].

Fifth, ambulatory and subcutaneous EEG monitoring hold promise for continuous, real-time surveillance of SWAS burden and treatment response [201]. Finally, adequately designed prospective studies with longitudinal neurodevelopmental follow-up into adulthood are needed to fully characterize the long-term burden of D/EE-SWAS.

## 8. Conclusions

D/EE-SWAS is a clinically heterogeneous and neurodevelopmentally consequential spectrum of childhood epileptic encephalopathies, for which traditional treatments have shown limited effectiveness in improving long-term cognitive outcomes. Its diagnosis requires sleep EEG with SWI quantification, high-resolution MRI, comprehensive genetic testing, and serial standardized neuropsychological assessment. Early diagnosis and treatment are critical: longer ESES duration during critical developmental windows drives irreversible cognitive sequelae.

Corticosteroids currently have the strongest available evidence for cognitive benefit, although the overall certainty of evidence remains low to moderate, and may therefore be considered first-line therapy. Benzodiazepines may be used as first- or second-line treatment. Other antiseizure medications that may be useful include levetiracetam, valproate, ethosuximide, and sulthiame. Surgery achieves the highest documented response rates in appropriately selected lesional or non-lesional focal cases and should be considered early in the management of drug-resistant D/EE-SWAS. Given the pervasive impact of D/EE-SWAS on neurodevelopmental trajectories, optimal management also extends beyond pharmacologic and surgical therapy to multidisciplinary care involving neurologists, neuropsychologists, rehabilitation specialists (speech-language, occupational, and physical therapy), educators, and genetic counselors. Most importantly, the field is at a time of genuine paradigm shift: the growing identification of genetic causes opens the door to precision medicine approaches. The optimal future management strategy will combine syndrome-driven and etiology-driven approaches, individualized to each patient’s clinical presentation, genetic diagnosis, and treatment response—guided by a systematic diagnostic algorithm and monitored with objective neurocognitive and EEG biomarkers.

## Figures and Tables

**Table 1:** Literature search and study selection methods.

Component	Details
Review Type	Narrative literature review; no preregistered protocol, formal risk-of-bias assessment, or meta-analysis
Databases Searched	PubMed/MEDLINE, Embase, and Web of Science, searched from inception to 15 January 2026
Core Search Terms	(“electrical status epilepticus in sleep” OR “ESES” OR “continuous spikes and waves during sleep” OR “CSWS” OR “spike and wave activation in sleep” OR “SWAS” OR “DEE-SWAS” OR “EE-SWAS”) combined individually with: “epileptic encephalopathy,” “developmental encephalopathy,” “developmental and epileptic encephalopathy,” “cognitive regression,” “language regression,” “genetics,” “precision therapy,” “corticosteroids,” “surgery,” “thalamocortical,” “neuroimaging,” “outcome,” and “biomarker”; database-specific controlled vocabulary (MeSH, Emtree) applied where available
Supplementary Gene-Targeted Search	For each genetic cause identified through the core search as associated with DEE-SWAS, a supplementary PubMed search was performed using the individual gene name (e.g., “GRIN2A,” “CNKSR2,” “DLG4,” “KCNA2,” “SCN2A”) combined with “precision therapy,” “targeted treatment,” “gene therapy,” “antisense oligonucleotide,” “drug repurposing,” or the specific mechanistic target (e.g., “NMDA receptor,” “mTOR,” “L-serine”); these searches aimed to capture therapeutic data not indexed under DEE-SWAS terminology and spanned inception to 15 January 2026
Hand Searching	Reference lists of key publications and relevant reviews were manually searched; foundational historical references were included regardless of publication date
Language	English; non-English publications included if sufficient data were extractable from English abstracts
Study Types Included	Original research articles; case reports and case series; cohort and registry studies; clinical trials (all phases); narrative and systematic reviews
Populations Included	Children and adults with a clinical diagnosis of DEE-SWAS, EE-SWAS, or D/EE-SWAS (including earlier nomenclature: ESES, CSWS, Landau–Kleffner syndrome, atypical BECTS/SeLECTS with SWAS); experimental models (rodent, zebrafish, cellular, iPSC-derived) where relevant to pathophysiology or treatment
Outcome Domains	Electroclinical phenotype; molecular and network pathophysiology; structural and functional neuroimaging; genetic findings and precision therapeutic targets; responses to pharmacological, dietary, neuromodulatory, and surgical interventions; neurodevelopmental and seizure outcomes; emerging biomarkers
Exclusion Criteria	Publications mentioning ESES/CSWS/SWAS only incidentally within broader epilepsy cohorts without phenotypic or mechanistic detail specific to DEE-SWAS; conference abstracts without full-text publication when content was duplicated in a peer-reviewed article
Handling of Heterogeneous Terminology	Older studies using ESES or CSWS terminology were included when the electroclinical context—sleep-activated epileptiform activity with developmental regression or plateau—was consistent with current DEE-SWAS criteria; strict retrospective reclassification was not performed, and residual cohort heterogeneity is acknowledged as a limitation
Preprints and Early-Phase Data	Included when judged to add important mechanistic or translational insight; such findings are explicitly identified in the text and should be interpreted cautiously pending peer review and replication
Article Selection	Selection was based on novelty of findings and relevance to clinicians managing DEE-SWAS; larger cohorts and longitudinal studies were emphasized, with case reports used to illustrate rare or emerging manifestations. This is a narrative rather than a systematic or scoping review, and a PRISMA flow diagram was therefore not used; for transparency, 1158 records were initially identified across the searches described above, 358 full-text articles were assessed for eligibility, and 152 studies (contributing a total of 199 citations) were ultimately included. Selection and screening were performed by the author.
Data Synthesis	Data extracted and summarized qualitatively, organized thematically by section; no pooled quantitative analysis was performed
Key Limitations	Narrative (non-systematic) design with no preregistered protocol or formal risk-of-bias assessment; susceptibility to publication bias; potential cohort overlap across multicenter registry studies; heterogeneity in diagnostic thresholds (SWI cutoffs), outcome measures, and follow-up duration limits cross-study comparisons

**Table 2:** Key studies in D/EE-SWAS.

Author(s) & Year	Study Design	Country	N/Population	Follow-Up	EEG/Seizure Response	Cognitive/Behavioral Outcome
Antiseizure Medications						
Ohtsuka et al. (1992) [102]	Prospective (VAL)	Japan	N = 4 (total cohort = 46); children with ESES	NR	4/4 seizure response	Not assessed/not reported
Ribacoba et al. (1997) [103]	Case series (VAL + ETX)	Spain	N = 2; children with ESES	NR	2/2 EEG response; 2/2 seizure response	Not assessed/not reported
Inutsuka et al. (2006) [104]	Prospective (VAL, ETX, ACTH. BZD)	Japan	N = 15; children with ESES	NR	10/15 EEG; 10/15 seizure response	Not assessed/not reported
Larsson et al. (2012) [105]	RCT (crossover); LEV	Norway	N = 18; children with ESES	12 weeks	LEV vs. PBO: 44% vs. 8% SWI reduction; 9/18 > 50% SWI reduction	No significant cognitive benefit
Bjørnæs et al. (2013) [120]	RCT (crossover); LEV	Norway	N = 23; children with ESES	12 weeks	LEV vs. PBO: 40% vs. 17% SWI reduction	No significant effect on behavior, vigilance, or memory
Chen et al. (2014) LEV [106]	Retrospective; LEV	China (multicenter)	N = 73; children with ESES	Mean 19 mo	15/73 (21%) ≥ 50% SWI reduction; 33/52 (63%) seizure reduction or freedom	Better in idiopathic vs. symptomatic group
Grosso et al. (2014) [110]	Prospective; Lacosamide	Italy	N = 8; children with CSWS	6 months	5/8 EEG response; 2/3 seizure response	2/8 cognitive improvement
Sanchez Fernandez et al. (2012) [107]	Retrospective; Diazepam	USA	N = 29; children with CSWS/ESES	24 h	15/29 (52%) ≥ 50% SWI reduction; 47% mean SWI reduction	Not assessed/not reported
Sanchez Fernandez et al. (2013) [121]	Retrospective; Diazepam	USA	N = 29; children with CSWS/ESES	4 wk/6 mo	8/29 (28%) ≥ 50% SWI reduction; 14/29 (48%) seizure-free	14/29 (48%) language; 11/29 (38%) cognitive; 4/29 (14%) behavioral improvement
Francois et al. (2014) [108]	Retrospective; Diazepam	USA	N = 42; children with ESES	10 mo–2 yr	18/26 (69%) > 50% SWI reduction; 65% mean SWI reduction	8/17 (47%) problem-solving; 5/11 speech; 4/11 writing improvement
Fejerman et al. (2012) [122]	Retrospective; Sulthiame	Argentina	N = 53; focal epilepsies with ESES	1.5–16 yr	15/53 (28%) EEG normalization; 31/53 (58%) seizure freedom	Seizure freedom/reduction associated with cognitive improvement
Topçu et al. (2021) [111]	Retrospective; Sulthiame	Turkey	N = 39; children with ESES	32.5 ± 13.7 months	28/39 EEG response; 33/39 seizure response	Not assessed/not reported
Kanmaz et al. (2021) [123]	Retrospective; Sulthiame	Turkey	N = 29; children with ESES	5 mo–4 yr	6/29 (21%) EEG normalization; 8/19 (42%) seizure-free	11/29 (38%) cognitive/behavioral improvement
Vrielynck et al. (2017) [109]	Retrospective; Topiramate	Belgium	N = 21; children with CSWS	12 mo	10/21 (48%) improved EEG grade; 6/13 (46%) seizure-free	9/10 EEG responders showed cognitive/behavioral improvement
Chen et al. (2015) LEV [124]	Retrospective; LEV	China	N = 71; children with ESES	3 mo–6.3 yr	24/71 (34%) SWI normalization; 25/50 (50%) seizure-free	13/71 (18%) cognitive recovery; 11/71 (15%) > 50% improvement
Wilson et al. (2018) [112]	Retrospective; Amantadine	USA	N = 20; refractory ESES	Median 11.5 mo	47% median SWI reduction	11/16 (69%) language, cognitive, or behavioral improvement
Corticosteroids and ACTH						
Marescaux et al. (1990) [113]	Case series; Hydrocortisone + Prednisone	France	N = 5; children with LKS	NR	3/3 EEG response; 3/3 seizure response	3/3 cognitive improvement
Buzatu et al. (2009) [114]	Retrospective; Hydrocortisone	Belgium	N = 44; children with ESES/CSWS	21 months	21/44 EEG normalization; 33/41 complete seizure control	37/44 (84%) cognitive improvement
Altunel et al. (2017) [27]	Retrospective; ACTH	Turkey	N = 75; children with ESES	6–15 days tx/13 mo follow-up	59% mean SWI reduction; 24/42 (57%) seizure-free	67% improvement in ADHD-like symptoms
Altunel et al. (2017) [125]	Retrospective; ACTH	Turkey	N = 25; children with ESES, ADHD/ASD, and stuttering	6–10 days tx/13 mo follow-up	51% median SWI reduction; 7/10 (70%) seizure-free	72% improvement in ADHD/ASD symptoms; stuttering resolved in 12/25 (48%)
Chen et al. (2014) [106]	Retrospective; MP + Prednisone	China	N = 82; children with ESES	NR	68/82 EEG response: 50–60% recurrence rate at 1 year	Not assessed/not reported
Chen et al. (2016) [126]	Prospective OL; Dexamethasone	China	N = 15; refractory CSWS	6–10 mo	5/15 (33%) ≥ 50% SWI reduction; 3/15 (20%) seizure-free	7/15 (47%) cognitive/behavioral improvement or resolution
Hempel et al. (2019) [117]	Prospective; Pulse Prednisone	USA	N = 17; children with ESES	Mean 10 mo	—	10(59%) had either language or behavior improvement. Trend toward language/behavioral improvement with ESES resolution
Cao et al. (2019) [115]	Prospective OL; MP + Prednisone	China	N = 22; children with CSWS	12 months	7/22 (32%) ≥ 50% seizure reduction; 4/22 (18%) seizure-free	IQ significantly higher in patients with ≥50% seizure reduction (*p* < 0.05)
van Arnhem et al. (2024)—RESCUE ESES [118]	RCT; Steroids vs. Clobazam	Multicenter (Europe)	N = 45 (steroids = 22, clobazam = 23)	6 months	Favor steroids for EEG	5/20 (25%) steroids vs. 0/18 (0%) clobazam showed ≥11.25 IQ point improvement
van Arnhem et al. (2026) [127]	Retrospective; Steroids vs. Clobazam	Multicenter (Europe)	N = 72 (steroids = 24, clobazam = 48)	6 months	Steroid led to greater SWI reduction	Steroid better in daily functioning (84% vs. 51%)
Observational Studies (Multi-Drug/Natural History)						
Arhan et al. (2015) [128]	Retrospective observational	Turkey	N = 59; children with ESES	Mean 4.5 yr	18/59 (31%) EEG normalization and seizure-free; 32/59 (54%) > 50% reduction	Cognitive improvement in pts with >75% seizure reduction; longer ESES correlated with worse cognition
Caraballo et al. (2013) [62]	Retrospective observational; unilateral PMG	Argentina	N = 43 with D/EE-SWAS (of 66 total)	Mean 13.5 yr	3/43 (7%) seizure freedom	Pts with >75% seizure reduction had improved IQ and school performance
Caraballo et al. (2013) [129]	Retrospective observational (multicenter)	Argentina/Italy	N = 117; children with D/EE-SWAS	2–22 yr	45/117 (38%) seizure-free; 91/117 (78%) EEG abnormalities at follow-up	Cognitive improvement with >75% seizure reduction; better outcomes in idiopathic etiology
Caraballo et al. (2014) [130]	Retrospective observational; LKS	Argentina	N = 29; children with LKS	Mean 12 yr	26/29 (90%) seizure freedom	8/29 (28%) complete language recovery; earlier onset and longer ESES linked to worse language outcomes
Caraballo et al. (2015) [131]	Retrospective observational	Argentina	N = 17; children with D/EE-SWAS, unusual EEG	Mean 7.5 yr	3/17 (18%) seizure freedom; 13/17 (76%) persistent EEG abnormalities	Pts with >75% seizure reduction had improved school performance and IQ
Carvalho et al. (2020) [42]	Retrospective observational	Portugal	N = 38; children with D/EE-SWAS (CSWS)	Mean 10.6 mo	14/16 (88%) SWI reduction	12/16 (75%) detectable improvement in behavior and/or school performance
Degerliyurt et al. (2015) [132]	Retrospective observational	Turkey	N = 22; children with D/EE-SWAS	Mean 3.8 yr	15/22 (68%) EEG improvement	VIQ (not PIQ) significantly reduced at follow-up (*p* < 0.05)
Fatema et al. (2015) [133]	Retrospective observational	Bangladesh	N = 10; children with CSWS	2 wk–1 yr	7/10 (70%) > 50% SWI reduction	10/10 (100%) at least some seizure/behavioral improvement
Fortini et al. (2013) [134]	Retrospective observational; hemi-ESES	Argentina	N = 21; children with hemi-ESES	Mean 8 yr	8/21 (38%) SWI and seizure normalization	Favorable outcomes in pts with seizure freedom or >75% reduction; better in unilateral PMG
Gencpinar et al. (2016) [30]	Retrospective observational	Turkey	N = 44; children with ESES/CSWS	≥2 yr	18/36 (50%) EEG normalization; 16/36 (44%) seizure-free	10/22 (45%) favorable cognitive outcomes
Gong et al. (2018) [135]	Retrospective observational; MP	China	N = 21; children with CSWS	NR	MP significantly reduced HFOs/spikes (*p* < 0.05); greater reduction in genetic/unknown vs. structural etiology	HFOs more sensitive to treatment than spikes
Maltoni et al. (2016) [39]	Retrospective observational	Italy	N = 61; children with CSWS	Mean 7.3 yr	Higher SWI correlated with lower IQ (*p* < 0.05)	IQ, VIQ, and PIQ negatively correlated with SWI and longer ESES duration (*p* < 0.0001)
Raha et al. (2012) [136]	Retrospective observational	India	N = 14; children with ESES and related syndromes	0.5–8 yr	5/11 (45%) SWI reduction < 50% with prednisolone	7/11 (64%) improved seizure/cognitive outcomes
Saraf et al. (2020) [137]	Retrospective observational	India	N = 52; children with CSWS	≥1 yr	13/52 (25%) seizure-free; better in idiopathic vs. symptomatic (*p* = 0.03)	32/52 (61%) language improvement; ≥3 favorable EEG markers predicted better language outcomes
Sonnek et al. (2021) [84]	Retrospective observational	Germany	N = 95; children with CSWS or near-CSWS	NR	Significant EEG improvement with steroids (*p* < 0.01) and surgery (*p* < 0.05)	30% of treatments associated with cognitive improvement
van den Munckhof et al. (2018) [138]	Retrospective observational	Netherlands	N = 47; children with D/EE-SWAS and cognitive deficits	Mean 13.1 mo	24% median SWI reduction; greater in pts with cognitive improvement (*p* = 0.002)	45% of treatments resulted in cognitive improvement; SWI change correlated with IQ change (*p* = 0.014)
Wiwattanadittakul et al. (2020) [139]	Retrospective observational	USA	N = 33; children with SWI ≥ 85%	Median 33 mo	21/33 (64%) ESES resolution; 18/33 (55%) seizure-free	Seizure-free pts more likely to achieve ESES resolution (*p* = 0.003)
Yilmaz et al. (2014) [140]	Retrospective observational	Turkey	N = 14; children with ESES	2–4 yr	7/14 (50%) normal EEGs; 12/14 (86%) seizure-free	No correlation between ESES duration and IQ (*p* > 0.05)
Other Therapies (Diet, Surgery, Precision Medicine)						
Kelley & Kossoff (2016) [119]	Systematic review; Ketogenic Diet	USA (multicenter)	N = 38; children with DEE-SWAS	NR	53% EEG improvement; 41% > 50% seizure reduction	45% cognitive improvement
van den Munckhof et al. (2015) [141]	Pooled analysis; Surgery	International	N = 575; pooled analysis	NR	90% EEG improvement with surgery	90% cognitive improvement
Ma et al. (2024) [142]	Systematic review; Surgery	International	NR	NR	Hemispherectomy: 79.7% SWAS resolution; Focal resection: 78.6%; MST: 63.9%; Callosotomy: 8.3%	58–71% cognitive improvement with resective surgery
Juliá-Palacios et al. (2024) [143]	Phase 2A; L-serine (GRIN LoF)	Spain (multicenter)	N = 18; patients with GRIN LoF variants	NR	5/18 (28%) complete epileptic activity resolution	Improvements in adaptive behavior, motor function, and quality of life

Abbreviations: ACTH = adrenocorticotropic hormone; ADHD = attention deficit hyperactivity disorder; ASD = autism spectrum disorder; BZD: benzodiazepine(s); CSWS = continuous spike-wave during slow sleep; D/EE-SWAS = developmental/epileptic encephalopathy with spike-and-wave activation in sleep; EEG = electroencephalogram; ESES = electrical status epilepticus during sleep; ETX: ethosuximide; HFOs = high-frequency oscillations; IQ = intelligence quotient; LEV = levetiracetam; LKS = Landau–Kleffner syndrome; LoF = loss of function; mo = months; MP = methylprednisolone; NR = not reported; OL = open-label; PBO = placebo; PIQ = performance IQ; PMG = polymicrogyria; pts = patients; RCT = randomized controlled trial; SWI = spike-wave index; tx = treatment; VAL: valproate; VIQ = verbal IQ; wk = weeks; yr = years.

**Table 3:** Genetic causes of D/EE-SWAS and precision medicine approaches.

Gene/Locus	Mechanism	Associated Syndrome	Variant Type	Potential Precision Therapy
*GRIN2A (16p13.2)*	NMDA receptor GluN2A subunit	Epilepsy-aphasia spectrum, LKS, DEE-SWAS	GoF or LoF	LoF: L-serine (Phase 2A evidence); GoF: memantine, radiprodil (preclinical/clinical trial); IVIG for GRIN2A variants
*GRIN2B*	NMDA receptor GluN2B subunit	DEE-SWAS, DEE	LoF	L-serine (emerging evidence for GRIN LoF)
*KCNQ2 (20q13.33)*	Kv7.2 K^+^ channel	Neonatal epilepsy, DEE-SWAS	LoF	Sodium channel blockers (most effective for LoF); avoid KCO drugs
*KCNA2 (1p13.3)*	Kv1.2 K^+^ channel	DEE-SWAS, DEE	GoF	4-Aminopyridine (K^+^ channel blocker)
*SCN2A*	Nav1.2 Na^+^ channel	DEE-SWAS, DEE	GoF or LoF	GoF: sodium channel blockers; LoF: avoid sodium channel blockers
*CNKSR2 (Xp22.12)*	Scaffold protein	DEE-SWAS (X-linked)	LoF	Syndrome-driven (ethosuximide, sulthiame); immunotherapy in some
*SLC9A6 (Xq26.3)*	Na^+^/H^+^ exchanger	Christianson syndrome, DEE-SWAS	LoF	Supportive; felbamate (case report), ketogenic diet
*CDKL5 (Xp22.13)*	Ser/Thr kinase	CDKL5 deficiency disorder, DEE-SWAS	LoF	Ganaxolone (FDA-approved for CDKL5), fenfluramine
*mTOR pathway (TSC1/TSC2*, *DEPDC5*, etc.)	mTOR hyperactivation	Focal cortical dysplasia type II, DEE-SWAS	GoF (pathway)	Everolimus/rapamycin (mTOR inhibitors)
*MECP2 (Xq28)*	Methyl CpG binding protein	Rett syndrome, DEE-SWAS	LoF	Trofinetide, gene therapy (investigational)
*RARS2 (6q15)*	Mitochondrial Arg-tRNA synthetase	PCH6, DEE-SWAS	LoF	Vatiquinone (investigational)
*ZEB2 (2q22.3)*	Transcription factor	Mowat–Wilson syndrome, DEE-SWAS	LoF	Syndrome-driven
*KANSL1 (17q21.31) del17q21.31*	Histone acetyltransferase	Koolen–de Vries syndrome, DEE-SWAS	Haploinsuff.	Ketogenic diet (case reports); syndrome-driven
*Dup15q (15q11-q13)*	UBE3A overexpression/copy gain	Dup15q syndrome, DEE-SWAS (35.7%)	Copy gain	Retinoic acid, ASO to reduce UBE3A levels (investigational); basmisanil (terminated)
*DLG4*	Scaffold protein at postsynaptic density	DLG4-related synaptopathy, DEE-SWAS (>25%)	LoF	Syndrome-driven

Abbreviations: ASO = antisense oligonucleotide; GoF = gain of function; LoF = loss of function; NMDA = N-methyl-D-aspartate; PCH6 = pontocerebellar hypoplasia type 6. Note: Many precision therapy recommendations are extrapolated from other developmental and epileptic encephalopathy syndromes and have not been specifically validated in controlled DEE-SWAS cohorts.

**Table 4:** Prognostic factors in DEE-SWAS.

Domain	Unfavorable Factors	Favorable Factors
Clinical	Age < 6 years at ESES onset; longer ESES/CSWS duration; earlier seizure onset; multiple seizure types; pre-existing neurodevelopmental disorder (DEE-SWAS subtype)	Age > 8 years at onset; shorter ESES duration; SeLECTS phenotype at onset; normal development before CSWS; later onset of aphasia (in LKS)
EEG	SWI ≥ 85%; high spike-wave frequency (SWF > 25/min); frontal EEG predominance; disrupted sleep architecture; persistent HFOs after treatment; bilateral SWI distribution	Normal EEG background; low SWF; absence of frontal spikes; generalized (not focal) spikes; SWI improvement with therapy; ≥3 favorable EEG markers
Etiology/Imaging	Structural lesion on MRI; thalamic injury; polymicrogyria; genetic encephalopathy (DEE-SWAS subtype); metabolic etiology; reduced thalamic volume	Normal MRI; idiopathic/non-lesional etiology; EE-SWAS subtype (normal prior development); surgical candidacy (resectable lesion)
Treatment Response	Drug resistance; polypharmacy; failure of steroids and benzodiazepines; relapse after initial remission; frontal epileptiform discharges on EEG (increased relapse risk)	Response to steroids or benzodiazepines; early EEG normalization; seizure freedom; later onset and concomitant benzodiazepine therapy (associated with higher steroid response)
Cognitive Outcome	IQ decline correlates with SWI; verbal IQ more impaired than performance IQ; longer ESES duration linked to residual deficits; impaired overnight slow-wave slope decrease at epileptic focus	Normal development before CSWS; cognitive improvement correlates with EEG improvement and seizure control; post-remission normalization of slow-wave slope predicts cognitive recovery

Abbreviations: CSWS = continuous spike-wave during slow sleep; EEG = electroencephalogram; ESES = electrical status epilepticus during sleep; HFOs = high-frequency oscillations; IQ = intelligence quotient; LKS = Landau–Kleffner syndrome; SeLECTS = self-limited epilepsy with centrotemporal spikes; SWF = spike-wave frequency; SWI = spike-wave index.

## Data Availability

No new data were created or analyzed in this study. Data sharing is not applicable to this article.
